# Unveiling heat vulnerability of older adults: an assessment of emergency hospital admissions in Switzerland

**DOI:** 10.1093/ije/dyag044

**Published:** 2026-04-08

**Authors:** Sujung Lee, Ana Maria Vicedo-Cabrera

**Affiliations:** Institute of Social and Preventive Medicine, University of Bern, Bern, Switzerland; Oeschger Centre for Climate Change Research, University of Bern, Bern, Switzerland; Institute of Social and Preventive Medicine, University of Bern, Bern, Switzerland; Oeschger Centre for Climate Change Research, University of Bern, Bern, Switzerland

**Keywords:** heat, vulnerability, emergency hospital admission, older adults, case time series

## Abstract

**Background:**

Older adults are highly vulnerable to heat, yet how individual characteristics modulate its effects remains unclear. We assessed heat-related emergency hospital admission (EHA) risk across subpopulations of older adults receiving home care services in Switzerland (2019–2022).

**Methods:**

We analysed patient-level EHA data linked to daily maximum temperature by MedStat regions of residence. We employed a case time series design with quasi-Poisson regression and distributed lag non-linear models to examine heat-related EHA risk, stratifying by individual characteristics, including sociodemographic factors, pre-existing health conditions, primary diagnosis, and levels of dependency and social interaction.

**Results:**

The overall heat-related EHA risk was 1.12 (95% confidence interval (CI): 1.04–1.20) (at 99th percentile vs minimum temperature percentile risk). Males (1.16; 1.04–1.29) generally showed higher heat-related EHA risk than females (1.09; 0.98–1.20), except among those aged ≥85 years (females 1.16; 1.00–1.34 vs males 1.06; 0.90–1.26). Regarding functional capacity, females requiring assistance with daily tasks had an increased heat-related EHA risk, whereas males showed the opposite trend, with higher risk among those who were independent. Sex-specific analyses revealed that anxiety and dementia/Alzheimer’s disease were risk factors for females, whereas cancer, chronic obstructive pulmonary disease, and coronary heart disease were risk factors for males. Joint stratification by pre-existing health conditions and primary diagnosis showed that individuals with pre-existing cancer had higher risks of admission for circulatory, genitourinary, infectious, and endocrine/metabolic causes during heat exposure.

**Conclusion:**

Our results show that older individuals are not equally vulnerable to heat, underscoring the need for targeted public health interventions to protect high-risk older adults.

Key Messages

- We investigated how individual characteristics of older adults modulate the risk of heat-related emergency hospital admissions (EHA) and revealed distinct patterns depending on sociodemographic factors, pre-existing health status, and levels of dependency and social interaction.
- Heat-related EHA risk showed opposite patterns between sexes: in males, higher risk was associated with younger age (65–84 years) and being functionally independent, whereas in females, increased risk was observed in the oldest age group (85+ years) and among those requiring assistance with daily tasks.
- Our findings emphasise the importance of considering the complexity of individual characteristics, including pre-existing health conditions and functional abilities, to move beyond generic approaches and deliver targeted public health interventions for high-risk older adults.

## Introduction

Extreme heat events are increasing in frequency and intensity, posing significant global health risks [1–4]. However, these effects are unevenly distributed, with certain groups—such as older adults [5, 6], individuals with pre-existing conditions or disabilities [7, 8], low-income populations [6], urban residents [9], and outdoor workers [10]—being more vulnerable.

Older adults are consistently identified as highly susceptible to heat [3, 5]. For example, several large multi-location studies have shown a clear dose–response relationship between age and heat-related mortality risks and/or death rates [2, 11, 12]. Ageing reduces the body’s ability to regulate body temperature, triggering harmful physiological responses [13–15], while chronic health issues common in older adults can worsen with heat exposure, increasing the risk of hospitalisation or death [16, 17]. This is concerning, given the population’s ageing and the rising temperatures due to climate change. Specifically, by 2050, 23% of the global population aged 69+ will live in areas experiencing extreme heat above 37.5°C, up from 14% in 2020 [18]. Thus, a large multi-location study projected that ageing would increase heat-related mortality under climate change [19].

While the vulnerability of older adults is well recognised, less is known about the individual-level factors contributing to these risks. Research in Switzerland showed that the risks of heat-related emergency hospital admissions (EHA) varied by age, sex, and primary diagnosis [20], and that ageing has intensified heat-related mortality—a trend expected to continue as populations age and temperatures rise due to climate change [21]. To date, only a handful of epidemiological assessments have examined how individual characteristics modulate heat-health risks of more vulnerable populations. For example, a study in China found that older adults with declining physical and cognitive abilities and social isolation faced higher mortality during heatwaves [22]. This research gap likely stems from limited access to detailed individual-level data, as most studies rely on administrative health records that include only a limited set of variables. Therefore, alternative health data sources and advanced statistical techniques are needed to improve understanding of heat-health vulnerabilities, clarify the mechanisms by which heat affects human health, and support targeted public health interventions.

This study aimed to examine how individual characteristics of older adults modulate their vulnerability to heat, as reflected in increased risk of EHA, using a unique dataset on hospitalisations in Switzerland. Specifically, we applied cutting-edge methods in climate epidemiology to a novel dataset created by linking hospital records with health survey data collected from home care services. It allowed us to assess heat-EHA risks across a wide range of individual characteristics (e.g., pre-existing conditions, levels of dependency and social interaction) and provide robust estimates of who is more vulnerable to heat among older adults.

## Methods

### Study population

**Figure 1:**
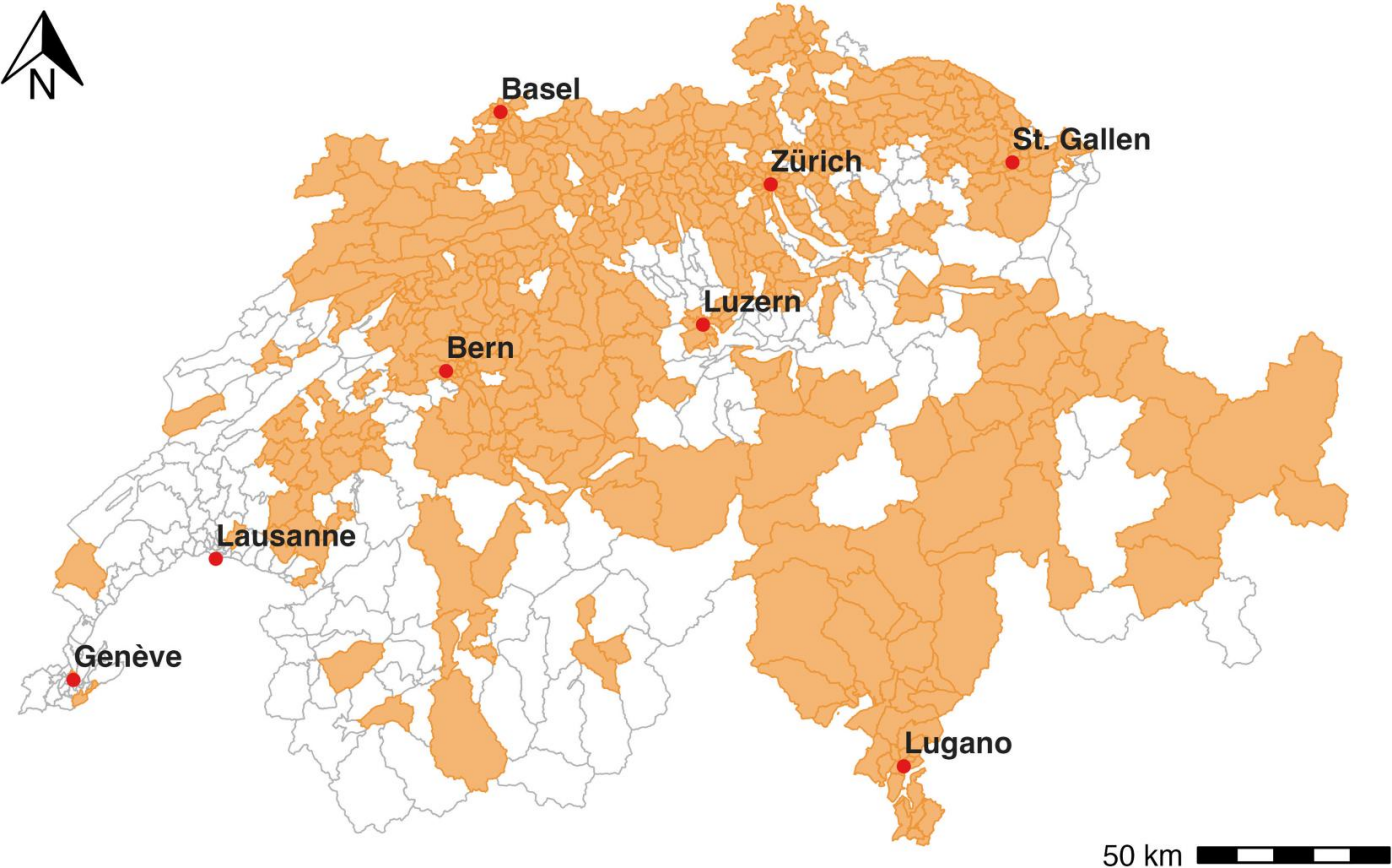
Spatial coverage of the study population. MedStat regions containing emergency hospital admission cases linked to survey data from the Spitex home care services are highlighted. Points indicate the locations of the eight main cities in Switzerland.

We collected individual-level EHA cases for Swiss residents aged 65+ receiving Spitex home care services between 2019 and 2022. The study covered 472 of 706 (67%) MedStat regions, a geographic unit used by the Swiss authorities to anonymise individual-level hospital admission data (Fig. 1). Proportions of the study population relative to the total population, along with their selection criteria, are provided in Supplementary Note 1.

### Health outcomes and individual-level information

The dataset included individual-level information, such as pre-existing health conditions, levels of social interaction, and autonomy in daily tasks. This dataset originated from HomeCareData (Spitex Schweiz, Bern) database and included all records registered between March 2020 and December 2023. The Federal Statistical Office linked this individual-level health information and EHA cases using unique patient identifiers created by SwissRDL (University of Bern) and assigned to both datasets. We linked the two datasets by matching each EHA case to the patient record that was closest in time, whether before or after the EHA case. We provide the detailed methods for merging the two datasets in Supplementary Note 2.

For pre-existing health conditions, we considered major chronic conditions (including cancer, cardiovascular, neurological, respiratory, and mental health disorders) and grouped them as either having documented conditions (labelled as ‘Yes’) or not (‘No’) (Supplementary Table S1).

We evaluated the functional status of individuals across three domains: activities of daily living in self-care (ADL—self-care) and mobility (ADL—mobility), and instrumental activities of daily living (IADL), categorising them as either requiring assistance or capable of performing tasks independently (Supplementary Table S1).

We assessed the levels of social interaction by participation in social activities, frequency of family visits and interactions with family members, and the amount of time spent alone. We classified the patients into high or low according to the standard in Supplementary Table S1.

We defined the primary diagnoses for EHA as the main reasons for the patient’s treatment and examination at discharge. We used the 10th revision of the International Classification of Diseases (ICD-10). We grouped the diagnoses into total EHA and major disease categories (Supplementary Table S1).

### Exposure assessment to heat

We obtained daily maximum temperatures on a 1-km grid across Switzerland from MeteoSwiss [23]. This gridded temperature data was generated by combining observations from various meteorological stations with an interpolation technique. We assigned each individual’s temperature exposure using the MedStat-level average temperature, matched to their Medstat of residence. To account for the heterogeneous spatial distribution of the population within MedStat regions due to irregular orography, we calculated population-weighted daily maximum temperatures using GPWv4 gridded population data (Revision11), resampled to 1-km resolution for 2020, following the method of a previous study [24, 25].

### Statistical analysis

We employed a case time series (CTS) design to evaluate transient health risks associated with time-varying exposures [26]. We constructed individual patient-level daily time series of temperature and EHA. To account for the diverse climate across Switzerland, we calculated Medstat-specific temperature percentiles, allowing us to express heat-related EHA risk on a relative temperature scale. We used conditional quasi-Poisson regression with distributed lag non-linear (DLNM) models over a 0–3-day lag, focusing on the warm season (May–September) to capture heat-related EHA risks.

We modelled the exposure-response dimension using a natural cubic spline with two internal knots at the 50th and 90th percentiles of the MedStat-specific warm-season temperature distribution. The lag-response dimension included an integer function, resulting in an unconstrained model with one parameter per lag day. Consistent with the CTS methodology, we defined the risk sets based on combinations of patient ID/month/year and included the day of the week as an explanatory variable. We also controlled for residual long-term trends using a natural cubic spline of time with 3 df per year. We selected model specifications based on established literature [20, 27] and the lowest q-AIC scores. Supplementary Table S2 summarises the tested parameters, including knot positions and functions.

To characterise distinct clinical phenotypes, we computed a dissimilarity matrix using Gower’s coefficient with the DAISY algorithm based on 16 pre-existing health conditions [28]. Hierarchical clustering was applied to this matrix to identify patient subgroups. This clustering procedure was performed separately for males and females. We subsequently conducted a stratified CTS analysis based on the identified clusters and primary diagnosis at discharge listed in the ‘Health outcomes and individual-level information’ section.

We reported our results for all models as relative risk (RR) at the 99th percentile of the Medstat-specific temperature distribution, with the minimum EHA risk temperature percentile (MHP) as the reference. The difference in risks between subgroups (e.g., males vs females) was reported as the ratio of RR (RRR) with corresponding 95% confidence intervals (CIs), following previous method [29]. We conducted statistical analyses using the R software (version 4.4.3) [30] with the *dlnm* [31] and *gnm* [32] packages.

## Results

### Descriptive statistics

Our analysis included 30 267 EHA cases among 19 010 older patients (aged ≥65 years) in Switzerland who received home care services from Spitex between 2019 and 2022 (Supplementary Table S3). Most admissions were due to non-external causes (71.8%), primarily circulatory diseases (18.2%), followed by digestive (8.9%) and respiratory diseases (7.1%). The patient population was predominantly Swiss (88.9%), female (55.7%), and aged 75 or older (82%). The most common pre-existing condition was cognitive impairment (48.1%), followed by coronary heart disease (32.8%) and diabetes (25%). Most patients received assistance with IADL (88.6%), and the proportions performing independently and receiving assistance were similar for ADL—self-care and ADL—mobility.

### Heat-EHA risks by sociodemographic characteristics

**Figure 2:**
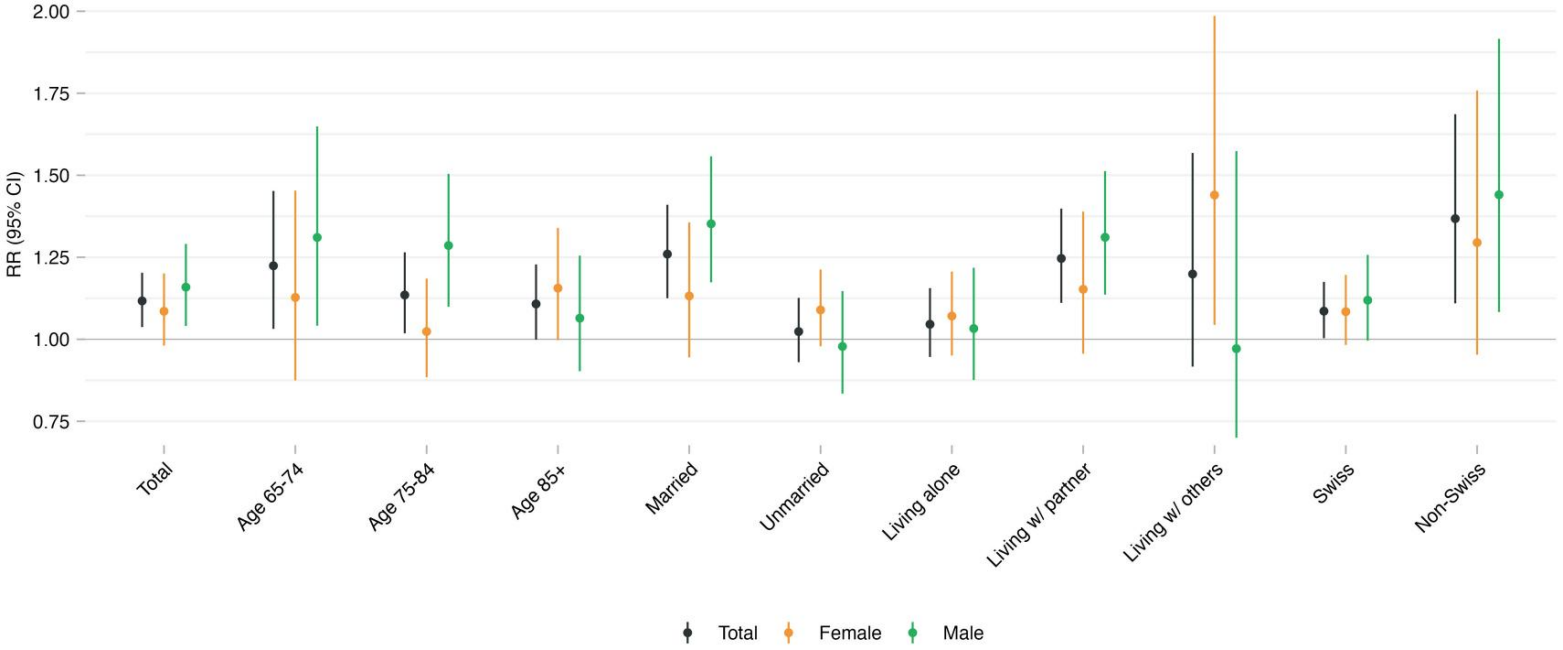
Overall and sex-specific heat-related emergency hospital admission risks by sociodemographic factors. Points represent cumulative relative risk (RR) at the 99th temperature percentile relative to the minimum hospitalisation risk percentile (MHP), with vertical lines indicating 95% confidence intervals (CIs). Estimates are provided for the total population (black) and stratified by females (yellow) and males (green).

Overall, the cumulative RR of heat-related hospitalisation, referred to as heat-EHA risk, was 1.12 (95% CI: 1.04–1.20) in the study population (Fig. 2). Heat-EHA risk decreased across age groups by sex. Males generally showed higher heat-EHA risks than females, both overall [males 1.16 (1.04–1.29) vs females 1.09 (0.98–1.20)] and across most age groups, except for individuals aged 85+ years, with a higher risk in females [1.16 (1.00–1.34) vs males 1.06 (0.90–1.26)]. Risks also varied by living arrangement. Among males, those living with a partner [1.31 (1.14–1.51)] had a higher risk than those in other living conditions, whereas among females, the highest risk was observed among those living with others (e.g., caregivers) [1.44 (1.04–1.99)]. However, except for the 75–84 age group, differences in risk between males and females were not significant (Supplementary Table S4).

### Impact of daily task capacity and social interaction on heat-EHA risks

**Figure 3:**
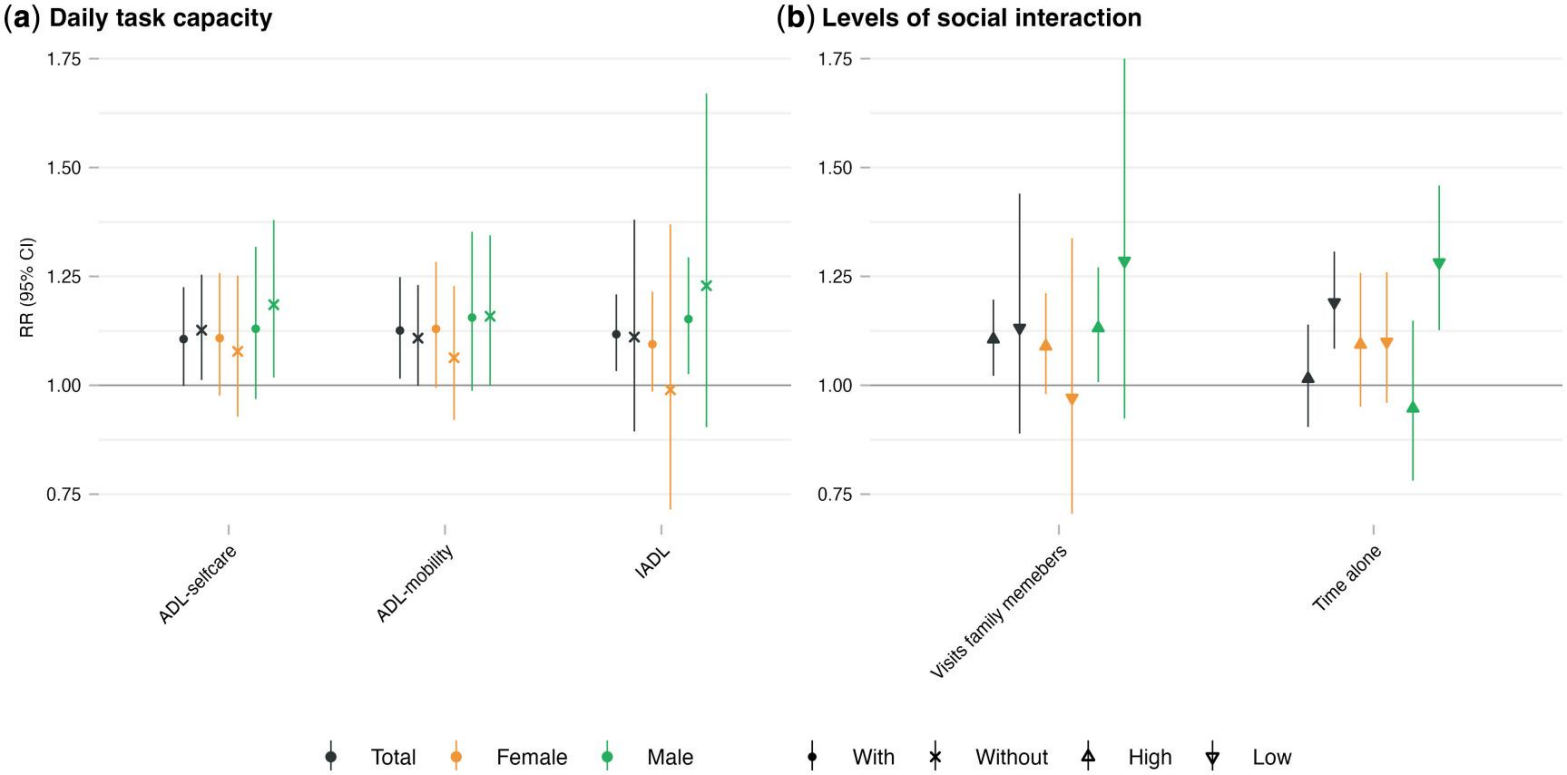
Sex-specific associations between heat exposure and emergency hospital admission risks, stratified by functional capacity and social integration. (a) Daily task capacity, assessed through activities of daily living (ADL—self-care and ADL—mobility), and instrumental activities of daily living (IADL). Symbols distinguish between individuals with (circle) and without (cross) assistance. (b) Levels of social interaction regarding family visits and time spent alone. Symbols distinguish between high (upward triangle) and low (downward triangle) levels. Points represent cumulative relative risk (RR) at the 99th temperature percentile relative to the minimum hospitalisation risk percentile (MHP), with 95% confidence intervals (CIs).

Figure 3 shows heat-EHA risks stratified by capacity for daily tasks and levels of social interaction. Overall, heat-EHA risk was similar between individuals who received assistance with mobility and daily activities and those who did not, with the latter showing a slightly lower estimate (Fig 3a). However, the pattern was reversed for self-care tasks: heat-EHA risk was higher among individuals who could perform self-care tasks independently [1.13 (1.01–1.25)] than among those who required assistance [1.11 (1.00–1.23)]. Notably, this pattern varied by sex: females had higher heat-EHA risks when receiving assistance with ADL—self-care [1.11 (0.98–1.26) vs 1.08 (0.93–1.25)], ADL—mobility [1.13 (0.99–1.28) vs 1.06 (0.92–1.23)], and IADL [1.09 (0.99–1.22) vs 0.99 (0.72–1.37)], whereas males showed similar or slightly higher risks when performing these tasks independently. Nevertheless, these findings were highly uncertain, as reflected by wide 95% CIs, which crossed the null value (RR = 1.00).

Figure 3b shows heat-EHA risk by levels of social interaction. Overall, we observed higher heat-EHA risk among those with less time alone [1.19 (1.08–1.31) vs high 1.02 (0.90–1.14)], with this effect mostly observed among males [1.28 (1.13–1.46)]. In contrast, the amount of time spent alone did not appear to affect heat-EHA risk in females. Females showed higher risk, though uncertain, with frequent visits to family members [1.09 (0.98–1.21)] compared to less frequent visits [0.97 (0.71–1.34)], whereas males had higher risk with less frequent visits [low 1.29 (0.92–1.79) vs high 1.13 (1.01–1.27)].

### Differences in heat-EHA risk by pre-existing health conditions and primary diagnosis at discharge

**Figure 4:**
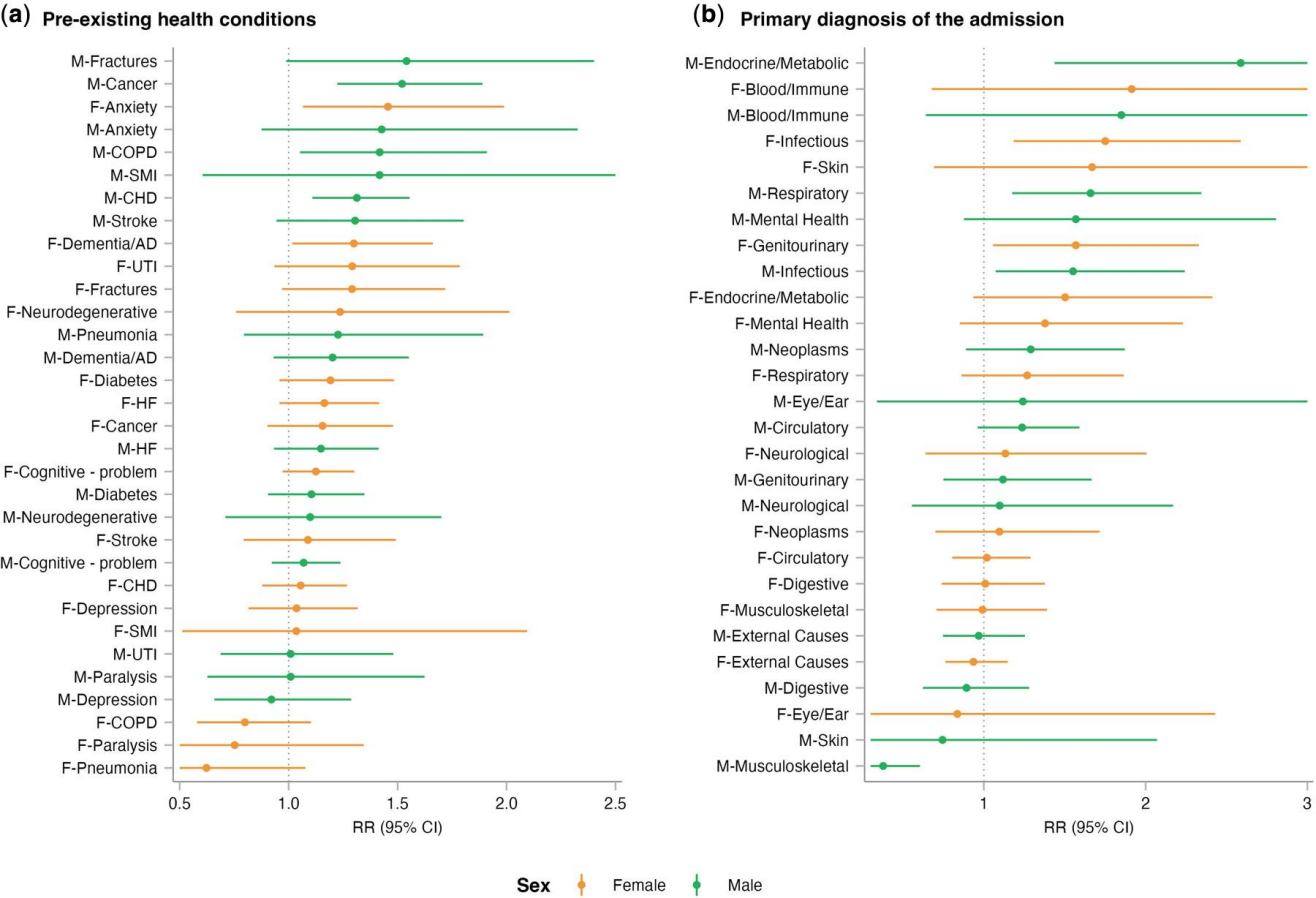
Sex-stratified heat-related emergency hospital admission risks by (a) pre-existing health conditions and (b) primary diagnosis. Points represent cumulative relative risk (RR) and 95% confidence intervals (CIs) at the 99th temperature percentile. Estimates are ranked in descending order of RR magnitude. CHD, coronary heart disease; HF, heart failure; SMI, severe mental illness; dementia/AD, dementia/Alzheimer’s disease; UTI, urinary tract infections.

Figure 4a presents sex-specific heat-EHA risk by pre-existing health conditions, ordered by cumulative RR at the 99th percentile to illustrate the risk gradient. Among males, higher risks were observed in those with cancer [1.52 (1.22–1.89)], chronic obstructive pulmonary disease (COPD) [1.42 (1.05–1.91)], and coronary heart disease [1.31 (1.11–1.56)]. In females, higher risks were noted for several mental health conditions, such as anxiety [1.46 (1.07–1.99)] and dementia/Alzheimer’s disease [1.30 (1.02–1.66)]. We observed a positive association for the other conditions, but the 95% CI included the null value (1.0), so the estimate remained uncertain.

Figure 4b shows heat-EHA risk by the primary diagnosis at discharge. Males admitted for endocrine/metabolic diseases reported the highest risk [2.59 (1.44–4.66)], followed by respiratory [1.66 (1.18–2.34)] and infectious [1.55 (1.07–2.24)] diseases, whereas a negative association was observed for musculoskeletal diseases [0.38 (0.24–0.61)]. Among females, we observed higher risks of admission due to infectious [1.75 (1.18–2.59)] and genitourinary [1.57 (1.06–2.33)] diseases.

**Figure 5:**
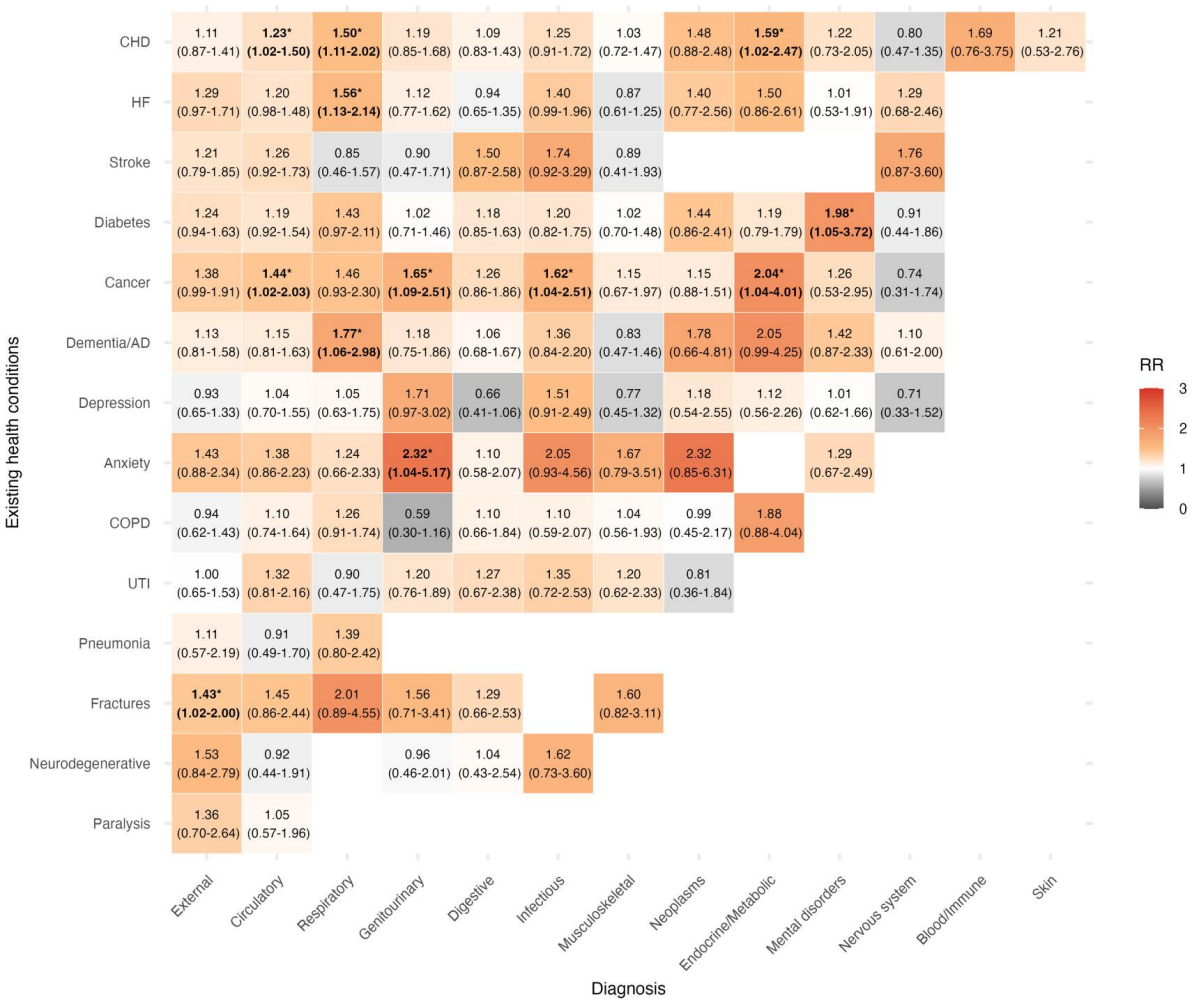
Heatmap of heat-related emergency hospital admission risks jointly stratified by pre-existing health conditions and primary diagnosis at discharge. Cell values represent cumulative relative risk (RR) and 95% confidence intervals (CIs) at the 99th temperature percentile. The colour gradient indicates the magnitude of RR, with darker orange representing higher risk. The results with the lower bound of the CI > 1.00 are marked with an asterisk (*). CHD, coronary heart disease; COPD, chronic obstructive pulmonary disease; HF, heart failure; SMI, severe mental illness; dementia/AD, dementia/Alzheimer’s disease; UTI, urinary tract infections.

Figure 5 presents heat-EHA risk jointly stratified by pre-existing health conditions and primary diagnoses at discharge. We first extracted cause-specific EHA cases and then stratified the analysis by including each pre-existing health condition as a binary indicator in the statistical model. Due to the limited number of cases, we did not perform a sex-stratified analysis. The highest risk was observed between pre-existing anxiety and genitourinary cause [2.32 (1.04–5.17)]. For individuals admitted due to respiratory diseases, pre-existing dementia/Alzheimer’s disease [1.77 (1.06–2.98)], heart failure [1.56 (1.13–2.14)], and coronary heart disease [1.50 (1.11–2.02)] were associated with increasing heat-EHA risk. For pre-existing cancer, we observed increasing heat-EHA risk among those admitted due to endocrine/metabolic [2.04 (1.04–4.01)], genitourinary [1.65 (1.09–2.51)], infectious [1.62 (1.04–2.51)], and circulatory [1.44 (1.02–2.03)] diseases.

Cluster analysis identified two distinct groups for each sex (Supplementary Fig. S2). Male-cluster1, characterised by a high prevalence of coronary heart diseases and heart failure, exhibited higher heat-EHA risks than male-cluster2. In females, female-cluster2 (distinguished by cognitive impairment and dementia/Alzheimer’s disease) showed elevated risk compared to female-cluster1. While we stratified the analysis by cluster and primary diagnosis at discharge, the results were inconclusive due to insufficient sample sizes (Supplementary Fig. S3).

## Discussion

We investigated how individual factors (pre-existing health conditions, functional capacity, and social interaction) affect heat-EHA risk. We identified sex-specific vulnerability profiles among older adults, with differential patterns across living conditions, functional status, and levels of social interaction, which allowed us to identify high-risk subgroups based on combinations of sex and specific health conditions.

Overall, older adults faced a 12% higher risk of EHA associated with heat exposure, consistent with previous findings from Switzerland [20] and elsewhere [33]. We observed differences in risk by sex: males had a 16% higher risk, while females had a 9% increase. However, among the oldest age group (85+ years), females showed a greater risk than males—a pattern aligned with heat-related mortality trends in Switzerland [34].

The results by primary diagnosis at discharge partially mirrored those of previous Swiss studies [20, 35–37], except for respiratory and circulatory diseases. We found a 46% higher risk of respiratory disease, consistent with Schulte *et al*. [20]; however, Ragettli *et al*. [36] reported no apparent increase in the summer of 2015. For circulatory diseases, our findings showed a non-significant positive association, which contrasts with Schulte *et al*. [38], who reported lower risks in older adults aged 75 years or older. These discrepancies may reflect differences in study populations. Our sample included older adults receiving home care services and accounted for approximately 3% of the total older adults aged 65+ in the selected MedStat regions. Thus, our study population may not be representative of the entire older adult population but is likely to be more vulnerable and to seek external support in the selected regions.

We observed an increased risk of heat-EHA across all functional status subgroups (with or without assistance), although the pattern varied by sex. Females had higher risk when dependent on assistance, while males had higher risk when independent. Nevertheless, estimates for some subgroups remained uncertain. Previous studies have shown that higher temperatures and longer daytime hours were associated with increased physical activity among older adults [39, 40]. We hypothesise that the higher risk among functionally independent individuals (reflecting the male risk patterns) is primarily attributable to greater heat exposure from increased outdoor activity. This exposure may override the protective effects of their better overall health. However, since data on outdoor activity were unavailable in this study, further research is required to confirm these mechanisms.

We observed that individuals living with someone else (e.g., a partner or caregivers) and spending less time alone faced higher heat-EHA risk, contrasting with previous studies that found social connections to be protective and living alone to increase heat-related hospitalisation risks [41, 42]. Similarly, studies on mortality have shown lower risk among socially engaged individuals [22, 43]. These divergent patterns suggest a complex role for social factors: higher social interaction or living with a partner/caregiver may lead to earlier or more frequent hospital visits but may also reduce mortality. Conversely, patients living alone would be more at risk of seeking support when the condition is more severe and possibly with a higher risk of mortality before being admitted.

Our stratified analysis of pre-existing health conditions found that males with cancer, COPD, and coronary heart disease, and females with anxiety and dementia/Alzheimer’s disease had higher EHA risks during heat exposure. These findings align with studies showing higher emergency admissions for those with chronic kidney disease or asthma/COPD [44]. To extend our analysis beyond single risk factors, we used clustering analysis to delineate holistic risk profiles. We identified distinct sex-specific susceptibilities, specifically a male profile characterised by pre-existing cardiovascular burden, and a female profile defined by pre-existing cognitive impairment. Furthermore, the joint stratification by pre-existing health conditions and primary diagnosis at discharge revealed distinct heat-EHA risk patterns; for example, pre-existing diabetes was linked to increased heat-EHA risk associated with mental health and respiratory diagnoses. This is consistent with prior research, including Lam *et al*. [45], who showed increased heat-related morbidity in vulnerable populations, specifically an increased risk of acute myocardial infarction among patients with diabetes. Additionally, pre-existing cancer was linked to an increased heat-EHA risk due to circulatory, genitourinary, infectious, and endocrine/metabolic causes. Patients with cancer frequently present with multimorbidity [46]; furthermore, chemotherapy can impair thermoregulation capacity and renal function [47]. This physiological compromise may increase susceptibility to acute heat stress, placing a burden on multiple organ systems.

We acknowledge several limitations. First, utilising residential information aggregated at the MedStat level may introduce exposure misclassification; however, this was mitigated by using population-weighted temperatures. Additionally, although we used temperature percentiles to account for local climatological differences, we were unable to capture other sources of spatial heterogeneity. Second, we were unable to assess socioeconomic status due to limited access to confidential data. Third, our findings were specific to older adults receiving home care services in Switzerland and may not generalise to all older adults. Fourth, temporal gaps between health survey data and admission could overlook intervening health or lifestyle changes. Finally, while the influence of COVID-19 cannot be entirely excluded, our restriction to the warm season (May–September)—a period with low transmission rates and no major public health restrictions in Switzerland [48]—suggests that residual confounding by the pandemic was minimal.

In conclusion, a key strength of our study lies in the use of novel, high-granularity individual-level data, including pre-existing health conditions, daily task capacity, and social interactions, which enabled subgroup profiling beyond general population estimates. Our findings reveal distinct patterns in heat-EHA risks among older adults, demonstrating that individual characteristics, including cause of admission and pre-existing health conditions, modulate the vulnerability. This evidence highlights that a ‘one-fits-all’ adaptation approach would be unsuitable and emphasises the need for targeted prevention strategies for the most vulnerable older adults.

## Ethics approval

Ethical approval was not required since all analyses were conducted using data collected from administrative sources, as per the relevant agreement with the parties.

## Supplementary Material

dyag044_Supplementary_Data

## Data Availability

The data were analysed under a user agreement with the Federal Statistical Office of Switzerland and cannot be made publicly available. However, a sample of the R code to reproduce the main analysis is provided in the GitHub repository of the corresponding author (https://github.com/anavica).

## References

[1] Gallo E , Quijal-Zamorano M, Méndez Turrubiates RF et al Heat-related mortality in Europe during 2023 and the role of adaptation in protecting health. Nat Med 2024;30:3101–5. 10.1038/s41591-024-03186-1 39134730

[2] Scovronick N , Sera F, Vu B, et al Temperature-mortality associations by age and cause: a multi-country multi-city study. Environ Epidemiol 2024;8:e336. 10.1097/EE9.0000000000000336 39323989 PMC11424137

[3] Ebi KL , Capon A, Berry P et al Hot weather and heat extremes: health risks. Lancet 2021;398:698–708. 10.1016/S0140-6736(21)01208-3 34419205

[4] Vicedo-Cabrera AM , Scovronick N, Sera F et al The burden of heat-related mortality attributable to recent human-induced climate change. Nat Clim Chang 2021;11:492–500. 10.1038/s41558-021-01058-x 34221128 PMC7611104

[5] Ballester J , Quijal-Zamorano M, Méndez Turrubiates RF et al Heat-related mortality in Europe during the summer of 2022. Nat Med 2023;29:1857–66. 10.1038/s41591-023-02419-z 37429922 PMC10353926

[6] Benmarhnia T , Deguen S, Kaufman JS et al Review article: vulnerability to heat-related mortality: a systematic review, meta-analysis, and meta-regression analysis. Epidemiology 2015;26:781–93. 10.1097/EDE.0000000000000375 26332052

[7] Kang Y , Baek I, Park J. Assessing heatwave effects on disabled persons in South Korea. Sci Rep 2024;14:3459. 10.1038/s41598-024-54015-x 38342943 PMC10859370

[8] Kenny GP , Sigal RJ, McGinn R. Body temperature regulation in diabetes. Temperature (Austin) 2016;3:119–45. 10.1080/23328940.2015.1131506 27227101 PMC4861190

[9] de Schrijver E , Royé D, Gasparrini A et al Exploring vulnerability to heat and cold across urban and rural populations in Switzerland. Environ Res Health 2023;1:025003. 10.1088/2752-5309/acab78 36969952 PMC7614344

[10] Venugopal V , Shanmugam R, Perumal Kamalakkannan L. Heat-health vulnerabilities in the climate change context—comparing risk profiles between indoor and outdoor workers in developing country settings. Environ Res Lett 2021;16:085008. 10.1088/1748-9326/ac1469

[11] Masselot P , Mistry MN, Rao S et al Estimating future heat-related and cold-related mortality under climate change, demographic and adaptation scenarios in 854 European cities. Nat Med 2025;31:1294–302. 10.1038/s41591-024-03452-2 39870815 PMC12003192

[12] Masselot P , Mistry M, Vanoli J, et al Excess mortality attributed to heat and cold: a health impact assessment study in 854 cities in Europe. Lancet Planet Health. 2023;7:e271–81, e281. 10.1016/S2542-5196(23)00023-2 36934727

[13] Balmain BN , Sabapathy S, Louis M et al Aging and thermoregulatory control: the clinical implications of exercising under heat stress in older individuals. Biomed Res Int 2018;2018:8306154. 10.1155/2018/8306154 30155483 PMC6098859

[14] Mora C , Counsell CWW, Bielecki CR, et al Twenty-seven ways a heat wave can kill you: deadly heat in the era of climate change. Circ Cardiovasc Qual Outcomes 2017;10:e004233. 10.1161/CIRCOUTCOMES.117.004233 29122837

[15] Kenney WL , Munce TA. Invited review: aging and human temperature regulation. J Appl Physiol (1985) 2003;95:2598–603. 10.1152/japplphysiol.00202.2003 14600165

[16] Layton JB , Li W, Yuan J, et al Heatwaves, medications, and heat-related hospitalization in older Medicare beneficiaries with chronic conditions. PloS One 2020;15:e0243665. 10.1371/journal.pone.0243665 33301532 PMC7728169

[17] Kenny GP , Yardley J, Brown C et al Heat stress in older individuals and patients with common chronic diseases. CMAJ 2010;182:1053–60. 10.1503/cmaj.081050 19703915 PMC2900329

[18] Falchetta G , De Cian E, Sue Wing I et al Global projections of heat exposure of older adults. Nat Commun 2024;15:3678. 10.1038/s41467-024-47197-5 38744815 PMC11094092

[19] Chen K , de Schrijver E, Sivaraj S, et al; MCC Collaborative Research Network. Impact of population aging on future temperature-related mortality at different global warming levels. Nat Commun 2024;15:1796. 10.1038/s41467-024-45901-z 38413648 PMC10899213

[20] Schulte F , Röösli M, Ragettli MS. Risk, attributable fraction and attributable number of cause-specific heat-related emergency hospital admissions in Switzerland. Int J Public Health 2024;69:1607349. 10.3389/ijph.2024.1607349 39435310 PMC11491377

[21] de Schrijver E , Bundo M, Ragettli MS et al Nationwide analysis of the heat- and cold-related mortality trends in Switzerland between 1969 and 2017: the role of population aging. Environ Health Perspect 2022;130:37001. 10.1289/EHP9835 35262415 PMC8906252

[22] Xi D , Liu L, Zhang M et al Risk factors associated with heatwave mortality in Chinese adults over 65 years. Nat Med 2024;30:1489–98. 10.1038/s41591-024-02880-4 38528168

[23] Federal Office of Meteorology and Climatology MeteoSwiss. *Spatial Climate Analyses.* https://www.meteoswiss.admin.ch/climate/the-climate-of-switzerland/spatial-climate-analyses.html (11 March 2026, date last accessed).

[24] de Schrijver E , Folly CL, Schneider R, et al A comparative analysis of the temperature‐mortality risks using different weather datasets across heterogeneous regions. GeoHealth 2021;5:e2020GH000363. 10.1029/2020GH000363 PMC8143899 34084982

[25] Earth Science Data Systems NASA. *Gridded Population of the World, Version 4 (GPWv4): Population Density, Revision 11.* https://www.earthdata.nasa.gov/data/catalog/sedac-ciesin-sedac-gpwv4-popdens-r11-4.11 (11 March 2026, date last accessed).

[26] Gasparrini A. The case time series design. Epidemiology 2021;32:829–37. 10.1097/EDE.0000000000001410 34432723 PMC7611753

[27] Requia WJ , Vicedo-Cabrera AM, de Schrijver E et al Association of high ambient temperature with daily hospitalization for cardiorespiratory diseases in Brazil: a national time-series study between 2008 and 2018. Environ Pollut 2023;331:121851. 10.1016/j.envpol.2023.121851 37211231

[28] Coombes CE , Liu X, Abrams ZB et al Simulation-derived best practices for clustering clinical data. J Biomed Inform 2021;118:103788. 10.1016/j.jbi.2021.103788 33862229 PMC9017600

[29] Altman DG , Bland JM. Interaction revisited: the difference between two estimates. BMJ 2003;326:219. 10.1136/bmj.326.7382.219 12543843 PMC1125071

[30] R Core Team. R: A Language and Environment for Statistical Computing. Vienna: R Foundation for Statistical Computing, 2025.

[31] Gasparrini A. Distributed lag linear and non-linear models in R: The Package dlnm. J Stat Soft 2011;43:1–20. PMC3191524 22003319

[32] Turner H , Firth D. *gnm: Generalized Nonlinear Models.* https://CRAN.R-project.org/package=gnm (11 March 2026, date last accessed).

[33] Alho AM , Oliveira AP, Viegas S, et al Effect of heatwaves on daily hospital admissions in Portugal, 2000–18: an observational study. Lancet Planet Health 2024;8:e318–26, e326. 10.1016/S2542-5196(24)00046-9 38729671

[34] Vicedo-Cabrera AM , de Schrijver E, Schumacher DL et al The footprint of human-induced climate change on heat-related deaths in the summer of 2022 in Switzerland. Environ Res Lett 2023;18:074037. 10.1088/1748-9326/ace0d0 38476980 PMC7615730

[35] Vicedo-Cabrera AM , Goldfarb DS, Kopp RE et al Sex differences in the temperature dependence of kidney stone presentations: a population-based aggregated case-crossover study. Urolithiasis 2020;48:37–46. 10.1007/s00240-019-01129-x 30900001 PMC7357996

[36] Ragettli MS , Vicedo-Cabrera AM, Flückiger B et al Impact of the warm summer 2015 on emergency hospital admissions in Switzerland. Environ Health 2019;18:66. 10.1186/s12940-019-0507-1 31412877 PMC6694501

[37] Bundo M , de Schrijver E, Federspiel A, et al Ambient temperature and mental health hospitalizations in Bern, Switzerland: A 45-year time-series study. PloS One 2021;16:e0258302. 10.1371/journal.pone.0258302 34637463 PMC8509878

[38] Schulte F , Röösli M, Ragettli MS. Heat-related cardiovascular morbidity and mortality in Switzerland: a clinical perspective. Swiss Med Wkly 2021;151:w30013. 10.4414/SMW.2021.w30013 34519460

[39] Klimek M , Peter RS, Denkinger M, et al; ActiFE Study Group. The relationship of weather with daily physical activity and the time spent out of home in older adults from Germany–the ActiFE study. Eur Rev Aging Phys Act 2022;19:6. 10.1186/s11556-022-00286-0 35151273 PMC8903592

[40] Wu YT , Luben R, Wareham N, et al Weather, day length and physical activity in older adults: cross-sectional results from the European Prospective Investigation into Cancer and Nutrition (EPIC) Norfolk Cohort. PloS One 2017;12:e0177767. 10.1371/journal.pone.0177767 28562613 PMC5451002

[41] Zhang Y , Nitschke M, Bi P. Risk factors for direct heat-related hospitalization during the 2009 Adelaide heatwave: a case crossover study. Sci Total Environ 2013;442:1–5. 10.1016/j.scitotenv.2012.10.042 23168533

[42] Zhang Y , Nitschke M, Krackowizer A, et al Risk factors of direct heat-related hospital admissions during the 2009 heatwave in Adelaide, Australia: a matched case–control study. BMJ Open 2016;6:e010666. 10.1136/bmjopen-2015-010666 PMC4893849 27256088

[43] Ragettli MS , Flückiger B, Vienneau D et al Vulnerability to heat-related mortality and the effect of prevention measures: a time-stratified case-crossover study in Switzerland. Swiss Med Wkly 2024;154:3410. 10.57187/s.3418 39463255

[44] Xu Z , Yi W, Bach A et al Multimorbidity and emergency hospitalisations during hot weather. EBioMedicine 2024;104:105148. 10.1016/j.ebiom.2024.105148 38705102 PMC11087953

[45] Lam HCY , Chan JCN, Luk AOY, et al Short-term association between ambient temperature and acute myocardial infarction hospitalizations for diabetes mellitus patients: a time series study. PLoS Med 2018;15:e1002612. 10.1371/journal.pmed.1002612 30016318 PMC6049878

[46] Fowler H , Belot A, Ellis L et al Comorbidity prevalence among cancer patients: a population-based cohort study of four cancers. BMC Cancer 2020;20:2. 10.1186/s12885-019-6472-9 31987032 PMC6986047

[47] Hassan AM , Nogueira L, Lin YL et al Impact of heatwaves on cancer care delivery: potential mechanisms, health equity concerns, and adaptation strategies. J Clin Oncol 2023;41:3104–9. 10.1200/JCO.22.01951 37098249

[48] Federal Office of Public Health. *Infectious Diseases Dashboard (IDD).* https://idd.bag.admin.ch/(11 March 2026, date last accessed).

