# Supplementary material for: Unveiling heat vulnerability of older adults: an assessment of emergency hospital admissions in Switzerland
Source: Int J Epidemiol. 2026 Apr 8;55(2):dyag044. doi: 10.1093/ije/dyag044 (PMC13064978; doi:10.1093/ije/dyag044)

**Supplementary Data** for “Unveiling heat vulnerability of older adults: an assessment of emergency hospital admissions in Switzerland”

*Sujung Lee^1,2^, Ana Maria Vicedo-Cabrera^1,2^*

1 Institute of Social and Preventive Medicine, University of Bern, Bern, Switzerland

2 Oeschger Centre for Climate Change Research, University of Bern, Bern, Switzerland

Table of Contents

[Supplementary Note 1. Characteristics of home care provision and study population coverage 2](#_Toc224136758)

[Supplementary Note 2. Data linkage and variable definition 3](#_Toc224136759)

[Supplementary Table S1. Definitions of key individual-level characteristics 4](#_Toc224136760)

[Supplementary Table S2. Summary of model parameters tested for exposure-response and lag-response dimensions 5](#_Toc224136761)

[Supplementary Table S3. Descriptive statistics of emergency hospital admissions (EHA) and the number of patients by category 6](#_Toc224136762)

[Supplementary Table S4. Sex-specific cumulative relative risk (RR) at the 99th temperature percentile and ratio of relative risk (RRR) 8](#_Toc224136763)

[Supplementary Figure S1. Overall heat-related EHA risks by demographic factors, primary diagnosis, and pre-existing health conditions 10](#_Toc224136764)

[Supplementary Figure S2. Profile of pre-existing health conditions across sex-specific clusters 11](#_Toc224136765)

[Supplementary Figure S3. Jointly stratified analysis of heat-related risks by sex-specific cluster and cause of admission 13](#_Toc224136766)

[Supplementary Figure S4. Sensitivity analysis with different knot positions for sociodemographic factors and primary diagnosis at discharge 14](#_Toc224136767)

# Supplementary Note 1. Characteristics of home care provision and study population coverage

1. **Home care eligibility** Access to Spitex home care services is established through two primary pathways: (1) direct initiation by the individual; or (2) referral by health-care professionals (e.g., physicians, hospital staff, or social workers), when home-based care is determined medically necessary, such as continuity of care following hospital discharge.
2. **National context** According to the Swiss Federal Statistical Office (FSO), approximately 450,000 individuals received Spitex services in 2023, representing nearly 5% of the national population [REF]. The majority of these recipients (83%; n = 377,015) were aged ≥65 years. Non-profit organisations account for > 70% of service delivery nationwide.
3. **Data source and study population.** We utilised the HomeCareData (HCD) database, a centralised registry that monitors service delivery across approximately 56% of all Spitex providers in Switzerland. To assess representativeness, we cross-referenced our data with FSO household and population statistics (STATPOP 2020). The final study population comprised 30,672 individuals aged ≥65 years who had emergency hospital admissions between January 2019 and December 2022. This sample represents 2.81% of the total older adult population in the selected MedStat regions.

# Supplementary Note 2. Data linkage and variable definition

1. **Longitudinal integration of home care assessment** Given the repeated-measures design of the Spitex data, we established a composite health profile for each individual to distinguish between chronic disease burden and dynamic functional status.
   1. **Comorbidities (cumulative prevalence approach)**: Pre-existing chronic conditions (e.g., diabetes, cancer) were defined using a cumulative history algorithm. A condition was classified as present for a specific time point if it was recorded in the current or any preceding survey. Once a diagnosis was documented, it was carried forward to all subsequent records (monotonic accumulation), ensuring the capture of the total historical disease burden regardless of reporting inconsistencies in later surveys.
   2. **Functional and social status (Time-dependent approach)**: Conversely, variables reflecting functional capacity (e.g., activities of daily living, mobility) and social interaction were treated as dynamic, time-dependent covariates. These values were not aggregated; instead, the data used in the analysis reflected the specific “snapshot” recorded at the time of the survey, capturing the patient’s status in close proximity to the hospital admission.
2. **Linkage with emergency hospital admission (EHA)** Integrated home care records were linked to EHA cases using unique, anonymous patient identifiers provided by the Swiss Federal Statistical Office (FSO). Linkage was performed based on temporal proximity. For each EHA case, we identified the single home care assessment that minimised the absolute time difference ($\left| t_{survey}-t_{admission} \right|$) relative to the admission date. This algorithm allowed linkage to the closest assessment, regardless of whether it occurred immediately preceding or following the hospital admission.

Supplementary Table S1. Definitions of key individual-level characteristics**. Abbreviations**: ADL, activities of daily living; IADL, instrumental activities of daily living.

| Category | Items | Classification |
| --- | --- | --- |
| Pre-existing health conditions | Cancer, diabetes, dementia/Alzheimer’s disease, stroke, coronary heart disease, congestive heart failure, fractures, paralysis, neurodegenerative, COPD, pneumonia, severe mental illness, depression, urinary tract infections. | - **Yes**: Patients who had a primary diagnosis, active treatment diagnoses, or diagnoses under observation without active treatment. - **No**: Patients for whom no diagnosis was recorded (diagnosis not available).   * **Note**: We created composite binary variables for neurodegenerative diseases (Parkinson’s disease, multiple sclerosis), severe mental illness (bipolar disorder, schizophrenia), and paralysis (hemiplegia, paraplegia, quadriplegia). These categories were coded as present ('Yes') if an individual had a diagnosis for any of the constituent conditions. |
| ADL -  Self-care | Bathing, eating, toilet use. | - **With assistance**: Conditions that required partial or full help with one or more tasks. - **Without assistance**: Conditions in which patients were able to perform all tasks independently. |
| ADL - Mobility | Locomotion indoors/outdoors, mobility in bed, walking. | - **With assistance**: Conditions required partial or full help with one or more tasks. - **Without assistance**: Conditions able to perform all tasks independently. |
| IADL | housework, meal preparation, medication management, shopping, using transport. | - **With assistance:** Conditions required partial or full help with one or more tasks. - **Without assistance:** Conditions able to perform all tasks independently. |
| Social interaction | Attending social activities, frequency of family visits, interactions with family members, time spent alone | - **High**: Social activity, family visits, and family interactions occurred within 30 days before the survey; time spent alone: 8 or more hours during the day. - **Low**: No interaction or interaction occurred >30 days ago; time spent alone: < 8 hours. |
| Main diagnosis | ICD-10 diagnosis codes for EHA representing the primary reason for patient’s treatment and examination | - Certain infectious and parasitic diseases (A00–B99) - Neoplasms (C00–C97) - Blood and immune disorders (D50–D89) - Endocrine, nutritional, and metabolic diseases (E00–E90) - Mental and behavioural disorders (F00–F99) - Diseases of the nervous system (G00–G99) - Diseases of the eye and ear (H00–H95) - Diseases of the circulatory system (I00–I99) - Diseases of the respiratory system (J00–J99) - Diseases of the digestive system (K00–K93) - Diseases of the skin and subcutaneous tissue (L00–L99) - Diseases of the musculoskeletal system and connective tissue (M00–M99) - Diseases of the genitourinary system (N00–N99) - Injury, poisoning, and certain other consequences of external causes (S00–T98). |

Supplementary Table S2. Summary of model parameters tested for exposure-response and lag-response dimensions**.** Parameters selected for the main model, determined by the lowest q-AIC score, is highlighted in bold.

| **Lag** | **Exposure-response** | **Lag-response** |
| --- | --- | --- |
| **3 days** | - Natural cubic spline at one knot (50^th^) - Natural cubic spine at one knot (90^th^) - **Natural cubic spline at two knots (50, 90^th^)** - Natural cubic spline at three knots (10, 50, 90^th^) - Natural cubic spline at three knots (10, 75, 90^th^) | Natural cubic spline with 1 knot placed at lag day 1 |
|  |  | Step function (strata) with two intervals: lags 0 and 1–3 (fun = "strata", breaks = 1) |
|  |  | **Unconstrained model with one parameter per lag day (fun = "integer").** |
| 5 days | - Natural cubic spline at one knot (50th) - Natural cubic spine at one knot (90th) - **Natural cubic spline at two knots (50, 90th)** - Natural cubic spline at three knots (10, 50, 90th) - Natural cubic spline at three knots (10, 75, 90th) | Natural cubic spline with 2 knots equally spaced on the log scale of lags. |
|  |  | 3 strata intervals at lag 0, 1, and 2-5 (fun = "strata", breaks = c(1,2)) |
|  |  | **Unconstrained model with one parameter per lag day (fun = "integer").** |
| 7 days | - Natural cubic spline at one knot (50th) - Natural cubic spine at one knot (90th) - **Natural cubic spline at two knots (50, 90th)** - Natural cubic spline at three knots (10, 50, 90th) - Natural cubic spline at three knots (10, 75, 90th) | Natural cubic spline with 2 knots equally spaced on the log scale of lags |
|  |  | 3 strata intervals at lags 0, 1-2, and 3-7 (fun = "strata", breaks = c(1,3)) |
|  |  | **Unconstrained model with one parameter per lag day (fun = "integer").** |

Supplementary Table S3. Descriptive statistics of emergency hospital admissions (EHA) and the number of patients by category**.** Unique patients counts are not stratified by primary diagnosis due to individuals having multiple admissions with different diagnoses. Percentage represent the proportion of the total study population, whereas gender distribution reflects the proportion of males and females within each specific category.

| **Category** | | **EHA (N, %)** | | | **Patients (N, %)** | | |
| --- | --- | --- | --- | --- | --- | --- | --- |
|  |  | **Total** | **Male** | **Female** | **Total** | **Male** | **Female** |
|  | Total | 30,267 (100%) | 13,954 (46.1%) | 16,313 (53.9%) | 19,010 (100%) | 8,427 (44.3%) | 10,583 (55.7%) |
| **Demographic** | |  |  |  |  |  |  |
|  | Age 65-74 | 5,823 (19.2%) | 3,170 (54.4%) | 2,653 (45.6%) | 3,515 (18.5%) | 1,853 (52.7%) | 1,662 (47.3%) |
|  | Age 75-84 | 12,761 (42.2%) | 6,081 (47.7%) | 6,680 (52.3%) | 8,039 (42.3%) | 3,709 (46.1%) | 4,330 (53.9%) |
|  | Age 85+ | 11,683 (38.6%) | 4,703 (40.3%) | 6,980 (59.7%) | 7,456 (39.2%) | 2,865 (38.4%) | 4,591 (61.6%) |
|  | Swiss | 26,694 (88.2%) | 11,986 (44.9%) | 14,708 (55.1%) | 16,904 (88.9%) | 7,329 (43.4%) | 9,575 (56.6%) |
|  | Non-Swiss | 3,573 (11.8%) | 1,968 (55.1%) | 1,605 (44.9%) | 2,106 (11.1%) | 1,098 (52.1%) | 1,008 (47.9%) |
|  | Married | 12,771 (42.2%) | 7,929 (62.1%) | 4,842 (37.9%) | 7,711 (40.6%) | 4,704 (61%) | 3,007 (39%) |
|  | Unmarried | 17,496 (57.8%) | 6,025 (34.4%) | 11,471 (65.6%) | 11,299 (59.4%) | 3,723 (32.9%) | 7,576 (67.1%) |
|  | Living alone | 15,855 (52.4%) | 5,524 (34.8%) | 10,331 (65.2%) | 10,316 (54.3%) | 3,431 (33.3%) | 6,885 (66.7%) |
|  | Living w/ partner | 12,391 (40.9%) | 7,809 (63%) | 4,582 (37%) | 7,452 (39.2%) | 4,619 (62%) | 2,833 (38%) |
|  | Living w/ others | 2,021 (6.7%) | 621 (30.7%) | 1,400 (69.3%) | 1,242 (6.5%) | 377 (30.4%) | 865 (69.6%) |
| **Primary diagnosis** | |  |  |  |  |  |  |
|  | Infectious and parasitic diseases | 1,862 (6.2%) | 930 (49.9%) | 932 (50.1%) | - | - | - |
|  | Neoplasms | 1,749 (5.8%) | 959 (54.8%) | 790 (45.2%) | - | - | - |
|  | Haematological and immune disorders | 292 (1%) | 129 (44.2%) | 163 (55.8%) | - | - | - |
|  | Endocrine and metabolic diseases | 1,019 (3.4%) | 446 (43.8%) | 573 (56.2%) | - | - | - |
|  | Mental and behavioural disorders | 1,081 (3.6%) | 454 (42%) | 627 (58%) | - | - | - |
|  | Diseases of the nervous system | 873 (2.9%) | 387 (44.3%) | 486 (55.7%) | - | - | - |
|  | Diseases of eye and ear | 243 (0.8%) | 88 (36.2%) | 155 (63.8%) | - | - | - |
|  | Diseases of the circulatory system | 5,521 (18.2%) | 2,616 (47.4%) | 2,905 (52.6%) | - | - | - |
|  | Diseases of the respiratory system | 2,151 (7.1%) | 1,182 (55%) | 969 (45%) | - | - | - |
|  | Diseases of the digestive system | 2,692 (8.9%) | 1,238 (46%) | 1,454 (54%) | - | - | - |
|  | Diseases of the skin and subcutaneous tissue | 336 (1.1%) | 161 (47.9%) | 175 (52.1%) | - | - | - |
|  | Diseases of the musculoskeletal system | 2,081 (6.9%) | 798 (38.3%) | 1,283 (61.7%) | - | - | - |
|  | Diseases of the genitourinary system | 1,825 (6%) | 937 (51.3%) | 888 (48.7%) | - | - | - |
|  | Injury and external causes | 5,850 (19.3%) | 2,312 (39.5%) | 3,538 (60.5%) | - | - | - |
| **Daily tasks** | |  |  |  |  |  |  |
|  | ADL (Self-care) – with assistance | 15,838 (52.3%) | 6,934 (43.8%) | 8,904 (56.2%) | 9,920 (52.2%) | 4,194 (42.3%) | 5,726 (57.7%) |
|  | ADL (Self-care) – without assistance | 14,399 (47.6%) | 7,003 (48.6%) | 7,396 (51.4%) | 9,071 (47.7%) | 4,223 (46.6%) | 4,848 (53.4%) |
|  | ADL (mobility) – with assistance | 15,127 (50%) | 6,569 (43.4%) | 8,558 (56.6%) | 9,256 (48.7%) | 3,855 (41.6%) | 5,401 (58.4%) |
|  | ADL (mobility) – without assistance | 15,140 (50%) | 7,385 (48.8%) | 7,755 (51.2%) | 9,754 (51.3%) | 4,572 (46.9%) | 5,182 (53.1%) |
|  | IADL – with assistance | 26,814 (88.6%) | 12,063 (45%) | 14,751 (55%) | 16,826 (88.5%) | 7,280 (43.3%) | 9,546 (56.7%) |
|  | IADL – without assistance | 3,099 (10.2%) | 1,667 (53.8%) | 1,432 (46.2%) | 1,970 (10.4%) | 1,015 (51.5%) | 955 (48.5%) |
| **Social interaction** | |  |  |  |  |  |  |
|  | Family visit – high | 26,461 (87.4%) | 11,969 (45.2%) | 14,492 (54.8%) | 16,681 (87.7%) | 7,258 (43.5%) | 9,423 (56.5%) |
|  | Family visit – low | 2,930 (9.7%) | 1,512 (51.6%) | 1,418 (48.4%) | 1,791 (9.4%) | 886 (49.5%) | 905 (50.5%) |
|  | Time alone – high | 11,377 (37.6%) | 4,145 (36.4%) | 7,232 (63.6%) | 7,407 (39%) | 2,578 (34.8%) | 4,829 (65.2%) |
|  | Time alone – low | 18,667 (61.7%) | 9,687 (51.9%) | 8,980 (48.1%) | 11,472 (60.3%) | 5,783 (50.4%) | 5,689 (49.6%) |
| **Existing health conditions** | |  |  |  |  |  |  |
|  | Stroke | 3,057 (10.1%) | 1,606 (52.5%) | 1,451 (47.5%) | 1,878 (9.9%) | 948 (50.5%) | 930 (49.5%) |
|  | Coronary heart disease | 9,932 (32.8%) | 5,358 (53.9%) | 4,574 (46.1%) | 5,899 (31%) | 3,074 (52.1%) | 2,825 (47.9%) |
|  | Herat failure | 7,381 (24.4%) | 3,715 (50.3%) | 3,666 (49.7%) | 4,334 (22.8%) | 2,093 (48.3%) | 2,241 (51.7%) |
|  | COPD | 3,400 (11.2%) | 1,901 (55.9%) | 1,499 (44.1%) | 1,905 (10%) | 1,062 (55.7%) | 843 (44.3%) |
|  | Anxiety | 2,079 (6.9%) | 607 (29.2%) | 1,472 (70.8%) | 1,242 (6.5%) | 339 (27.3%) | 903 (72.7%) |
|  | SMI | 483 (1.6%) | 182 (37.7%) | 301 (62.3%) | 304 (1.6%) | 112 (36.8%) | 192 (63.2%) |
|  | Depression | 3,808 (12.6%) | 1,349 (35.4%) | 2,459 (64.6%) | 2,312 (12.2%) | 792 (34.3%) | 1,520 (65.7%) |
|  | Paralysis | 1,042 (3.4%) | 627 (60.2%) | 415 (39.8%) | 645 (3.4%) | 371 (57.5%) | 274 (42.5%) |
|  | Neurodegenerative | 1,464 (4.8%) | 878 (60%) | 586 (40%) | 888 (4.7%) | 525 (59.1%) | 363 (40.9%) |
|  | Pneumonia | 1,258 (4.2%) | 723 (57.5%) | 535 (42.5%) | 716 (3.8%) | 405 (56.6%) | 311 (43.4%) |
|  | UTI | 2,420 (8%) | 1,038 (42.9%) | 1,382 (57.1%) | 1,393 (7.3%) | 569 (40.8%) | 824 (59.2%) |
|  | Cancer | 6,138 (20.3%) | 3,457 (56.3%) | 2,681 (43.7%) | 3,657 (19.2%) | 2,019 (55.2%) | 1,638 (44.8%) |
|  | Diabetes | 7,558 (25%) | 4,197 (55.5%) | 3,361 (44.5%) | 4,509 (23.7%) | 2,445 (54.2%) | 2,064 (45.8%) |
|  | Fractures | 2,488 (8.2%) | 834 (33.5%) | 1,654 (66.5%) | 1,598 (8.4%) | 483 (30.2%) | 1,115 (69.8%) |
|  | Cognitive problems | 14,559 (48.1%) | 6,818 (46.8%) | 7,741 (53.2%) | 9,199 (48.4%) | 4,109 (44.7%) | 5,090 (55.3%) |
|  | Dementia/Alzheimer’s disease | 4,688 (15.5%) | 2,002 (42.7%) | 2,686 (57.3%) | 3,061 (16.1%) | 1,268 (41.4%) | 1,793 (58.6%) |

Supplementary Table S4. Sex-specific cumulative relative risk (RR) at the 99th temperature percentile and ratio of relative risk (RRR)**.** All estimates are presented with 95% confidence intervals (CI). Differences between males and females for each category were calculated as the ratio of relative risk. Significant differences (lower CI bound > 1.00) are marked with *. **Abbreviations**: CHD, coronary heart disease; COPD, chronic obstructive pulmonary disease; HF, heart failure; SMI, severe mental illness; Dementia/AD, dementia/Alzheimer’s disease; UTI, urinary tract infections.

| **Category** | **RR-Female** | **RR-Male** | **RRR**  **(male vs. female)** |
| --- | --- | --- | --- |
| Total | 1.09 (0.98-1.20) | 1.16 (1.04-1.29) | 1.07 (0.92-1.24) |
| Age 65-74 | 1.13 (0.87-1.45) | 1.31 (1.04-1.65) | 1.16 (0.83-1.64) |
| Age 75-84 | 1.02 (0.88-1.18) | 1.29 (1.10-1.50) | **1.26 (1.01-1.56) *** |
| Age 85+ | 1.16 (1.00-1.34) | 1.06 (0.90-1.26) | 0.92 (0.74-1.15) |
| Married | 1.13 (0.94-1.36) | 1.35 (1.17-1.56) | 1.19 (0.95-1.50) |
| Unmarried | 1.09 (0.98-1.21) | 0.98 (0.83-1.15) | 0.90 (0.74-1.09) |
| Living alone | 1.07 (0.95-1.21) | 1.03 (0.88-1.22) | 0.96 (0.79-1.18) |
| Living w/ partner | 1.15 (0.96-1.39) | 1.31 (1.14-1.51) | 1.14 (0.90-1.44) |
| Living w/ others | 1.44 (1.04-1.99) | 0.97 (0.60-1.57) | 0.67 (0.38-1.21) |
| Swiss | 1.08 (0.98-1.20) | 1.12 (1.00-1.26) | 1.03 (0.89-1.20) |
| Non-Swiss | 1.29 (0.95-1.76) | 1.44 (1.08-1.92) | 1.11 (0.73-1.69) |
|  |  |  |  |
| Infectious | 1.75 (1.18-2.59) | 1.55 (1.07-2.24) | 0.89 (0.52-1.52) |
| Neoplasms | 1.10 (0.70-1.72) | 1.29 (0.89-1.87) | 1.18 (0.66-2.11) |
| Blood/Immune | 1.91 (0.68-5.40) | 1.85 (0.64-5.33) | 0.97 (0.22-4.29) |
| Endocrine/Metabolic | 1.50 (0.94-2.41) | 2.59 (1.44-4.66) | 1.72 (0.81-3.68) |
| Mental Health | 1.38 (0.85-2.23) | 1.57 (0.88-2.81) | 1.14 (0.53-2.43) |
| Neurological | 1.13 (0.64-2.01) | 1.10 (0.56-2.17) | 0.97 (0.40-2.37) |
| Eye/Ear | 0.84 (0.29-2.43) | 1.24 (0.34-4.54) | 1.48 (0.28-8.02) |
| Circulatory | 1.02 (0.80-1.29) | 1.24 (0.96-1.59) | 1.21 (0.86-1.72) |
| Respiratory | 1.27 (0.86-1.86) | 1.66 (1.18-2.34) | 1.31 (0.78-2.20) |
| Digestive | 1.01 (0.74-1.38) | 0.89 (0.62-1.28) | 0.88 (0.55-1.43) |
| Skin | 1.67 (0.69-4.03) | 0.74 (0.27-2.07) | 0.45 (0.12-1.73) |
| Musculoskeletal | 0.99 (0.71-1.39) | 0.38 (0.24-0.61) | **0.38 (0.21-0.68) *** |
| Genitourinary | 1.57 (1.06-2.33) | 1.12 (0.75-1.67) | 0.71 (0.41-1.25) |
| External Causes | 0.94 (0.76-1.15) | 0.97 (0.75-1.25) | 1.04 (0.74-1.44) |
|  |  |  |  |
| Cognitive - problem | 1.13 (0.97-1.30) | 1.07 (0.92-1.24) | 0.95 (0.77-1.17) |
| Cognitive - work | 1.05 (0.91-1.21) | 1.26 (1.09-1.47) | 1.20 (0.98-1.48) |
| Dementia/AD - No | 1.05 (0.94-1.17) | 1.16 (1.04-1.31) | 1.11 (0.95-1.31) |
| Dementia/AD - Yes | 1.30 (1.02-1.66) | 1.20 (0.93-1.55) | 0.92 (0.65-1.32) |
| Stroke - No | 1.09 (0.98-1.21) | 1.14 (1.02-1.28) | 1.05 (0.90-1.23) |
| Stroke - Yes | 1.09 (0.79-1.49) | 1.30 (0.94-1.80) | 1.20 (0.76-1.89) |
| CHD - No | 1.11 (1.00-1.24) | 1.09 (0.96-1.24) | 0.98 (0.83-1.16) |
| CHD - Yes | 1.06 (0.88-1.27) | 1.31 (1.11-1.56) | 1.24 (0.97-1.60) |
| HF - No | 1.07 (0.95-1.20) | 1.17 (1.03-1.32) | 1.09 (0.92-1.30) |
| HF - Yes | 1.16 (0.96-1.42) | 1.15 (0.93-1.41) | 0.99 (0.74-1.31) |
| Fractures - No | 1.07 (0.96-1.19) | 1.14 (1.02-1.27) | 1.06 (0.91-1.24) |
| Fractures - Yes | 1.29 (0.97-1.72) | 1.54 (0.99-2.40) | 1.19 (0.70-2.03) |
| Anxiety - No | 1.05 (0.94-1.17) | 1.15 (1.03-1.29) | 1.10 (0.94-1.28) |
| Anxiety - Yes | 1.46 (1.07-1.99) | 1.43 (0.88-2.33) | 0.98 (0.55-1.76) |
| SMI - No | 1.09 (0.98-1.20) | 1.16 (1.04-1.29) | 1.07 (0.92-1.24) |
| SMI - Yes | 1.04 (0.51-2.09) | 1.42 (0.60-3.32) | 1.37 (0.45-4.16) |
| Depression - No | 1.09 (0.98-1.22) | 1.19 (1.06-1.33) | 1.09 (0.93-1.27) |
| Depression - Yes | 1.04 (0.82-1.32) | 0.92 (0.66-1.29) | 0.89 (0.59-1.34) |
| Paralysis - No | 1.10 (0.99-1.22) | 1.17 (1.04-1.30) | 1.06 (0.91-1.24) |
| Paralysis - Yes | 0.75 (0.42-1.34) | 1.01 (0.63-1.62) | 1.34 (0.63-2.85) |
| Neurodegenerative - No | 1.08 (0.98-1.20) | 1.16 (1.04-1.30) | 1.07 (0.92-1.25) |
| Neurodegenerative - Yes | 1.24 (0.76-2.01) | 1.10 (0.71-1.70) | 0.89 (0.46-1.72) |
| COPD - No | 1.12 (1.01-1.25) | 1.13 (1.00-1.26) | 1.00 (0.86-1.17) |
| COPD - Yes | 0.80 (0.58-1.10) | 1.42 (1.05-1.91) | **1.77 (1.14-2.76) *** |
| Pneumonia - No | 1.11 (1.00-1.23) | 1.17 (1.04-1.30) | 1.05 (0.91-1.23) |
| Pneumonia - Yes | 0.62 (0.36-1.08) | 1.23 (0.79-1.89) | 1.97 (0.98-3.98) |
| UTI - No | 1.09 (0.98-1.21) | 1.18 (1.05-1.32) | 1.08 (0.93-1.26) |
| UTI - Yes | 1.29 (0.93-1.78) | 1.01 (0.69-1.48) | 0.78 (0.47-1.29) |
| Cancer - No | 1.08 (0.98-1.20) | 1.06 (0.94-1.20) | 0.98 (0.84-1.15) |
| Cancer - Yes | 1.16 (0.90-1.48) | 1.52 (1.22-1.89) | 1.32 (0.95-1.83) |
| Diabetes - No | 1.08 (0.98-1.20) | 1.18 (1.04-1.35) | 1.09 (0.93-1.29) |
| Diabetes - Yes | 1.19 (0.96-1.48) | 1.10 (0.91-1.35) | 0.93 (0.69-1.25) |
|  |  |  |  |
| Visits family members - High | 1.09 (0.98-1.21) | 1.13 (1.01-1.27) | 1.04 (0.89-1.22) |
| Visits family members - Low | 0.97 (0.71-1.34) | 1.29 (0.92-1.79) | 1.32 (0.84-2.10) |
| Time alone - High | 1.09 (0.95-1.26) | 0.95 (0.78-1.15) | 0.87 (0.68-1.10) |
| Time alone - Low | 1.10 (0.96-1.26) | 1.28 (1.13-1.46) | 1.17 (0.97-1.41) |
| ADL-selfcare - With | 1.11 (0.98-1.26) | 1.13 (0.97-1.32) | 1.02 (0.84-1.25) |
| ADL-selfcare - Without | 1.08 (0.93-1.25) | 1.19 (1.02-1.38) | 1.10 (0.89-1.36) |
| ADL-mobility - With | 1.13 (0.99-1.28) | 1.16 (0.99-1.35) | 1.02 (0.84-1.25) |
| ADL-mobility - Without | 1.06 (0.92-1.23) | 1.16 (1.00-1.34) | 1.09 (0.89-1.34) |
| IADL - With | 1.09 (0.99-1.22) | 1.15 (1.03-1.29) | 1.05 (0.90-1.23) |
| IADL - Without | 0.99 (0.72-1.37) | 1.23 (0.90-1.67) | 1.24 (0.79-1.95) |

Supplementary Figure S1. Overall heat-related EHA risks by demographic factors, primary diagnosis, and pre-existing health conditions**.** Cumulative relative risks (RR) and the 95% confidence intervals (CI) were calculated at the 99th temperature percentile relative to the minimum hospitalisation risk percentile (MHP). The MHP was determined specifically for each subgroup. **Abbreviations**: CHD, coronary heart disease; COPD, chronic obstructive pulmonary disease; HF, heart failure; SMI, severe mental illness; Dementia/AD, dementia/Alzheimer’s disease; UTI, urinary tract infections.


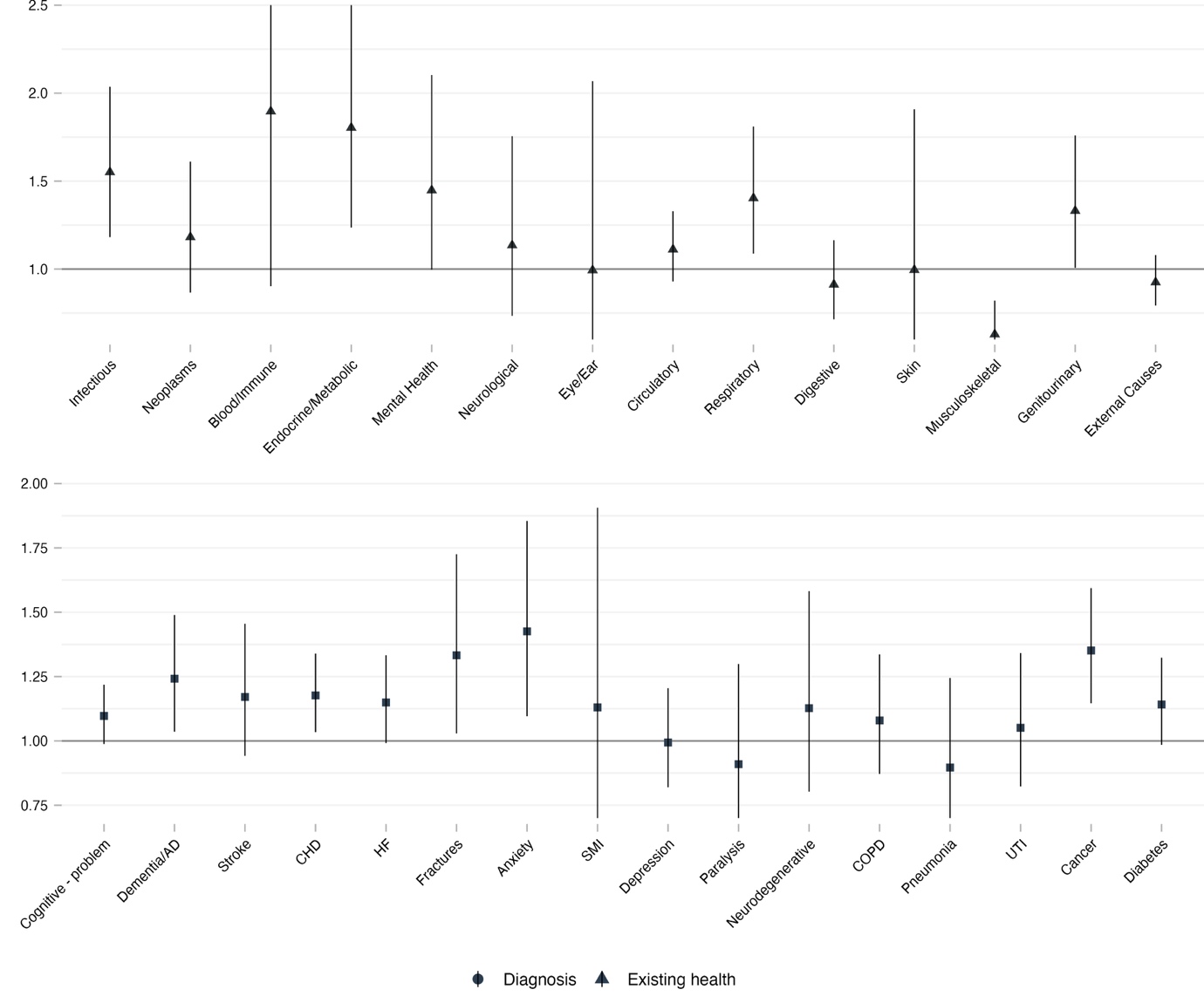


Supplementary Figure S2. Profile of pre-existing health conditions across sex-specific clusters**.** The radar plots display the prevalence (%) of each condition within the identified clusters for males and females. **Abbreviations**: CHD, coronary heart disease; COPD, chronic obstructive pulmonary disease; HF, heart failure; SMI, severe mental illness; Dementia/AD, dementia/Alzheimer’s disease; UTI, urinary tract infections.

**
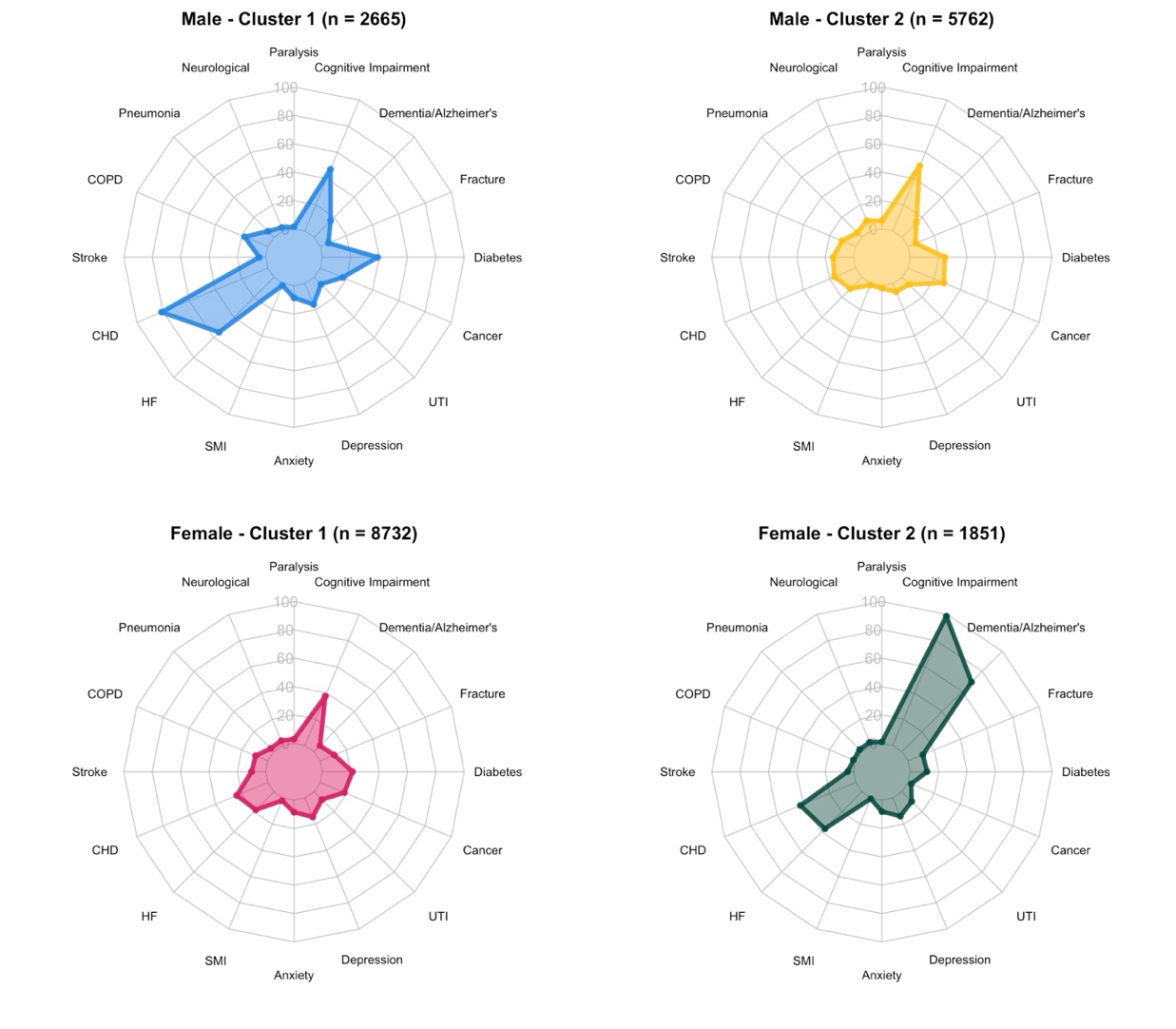
**

The characteristics of each cluster are:

- Male-Cluster 1 (n=2,665): Coronary heart disease and heart failure are the most prominent conditions in this group (around 60-80%), accompanied by moderate levels of cognitive impairment and diabetes. This pattern suggests a cardiovascular-dominant cluster with secondary metabolic and cognitive components.
- Male-Cluster 2 (n=5,762): This larger cluster presents a more even distribution across health conditions, with a moderate peak in cognitive impairment (~40%). Overall, it reflects a mixed-risk group with a generally lower overall disease burden.
- Female-Cluster 1 (n=8,732): This group shows generally low prevalence across most conditions, with only modest elevations in cognitive impairment (approximately 40%). The pattern suggests a broadly distributed health profile without a dominant condition, indicating a relatively lower-risk group.
- Female-Cluster 2 (n=1,851): There is a pronounced spike in cognitive impairment, dementia and Alzheimer’s (approaching 80-100%), indicating a subgroup with substantial neurological and cognitive burden. Other conditions occur at lower rates.

Supplementary Figure S3. Jointly stratified analysis of heat-related risks by sex-specific cluster and cause of admission**.** Cumulative relative risk (RR) and 95% confidence intervals (CI) were estimated for cause-specific admissions, stratified by sex-specific clusters.


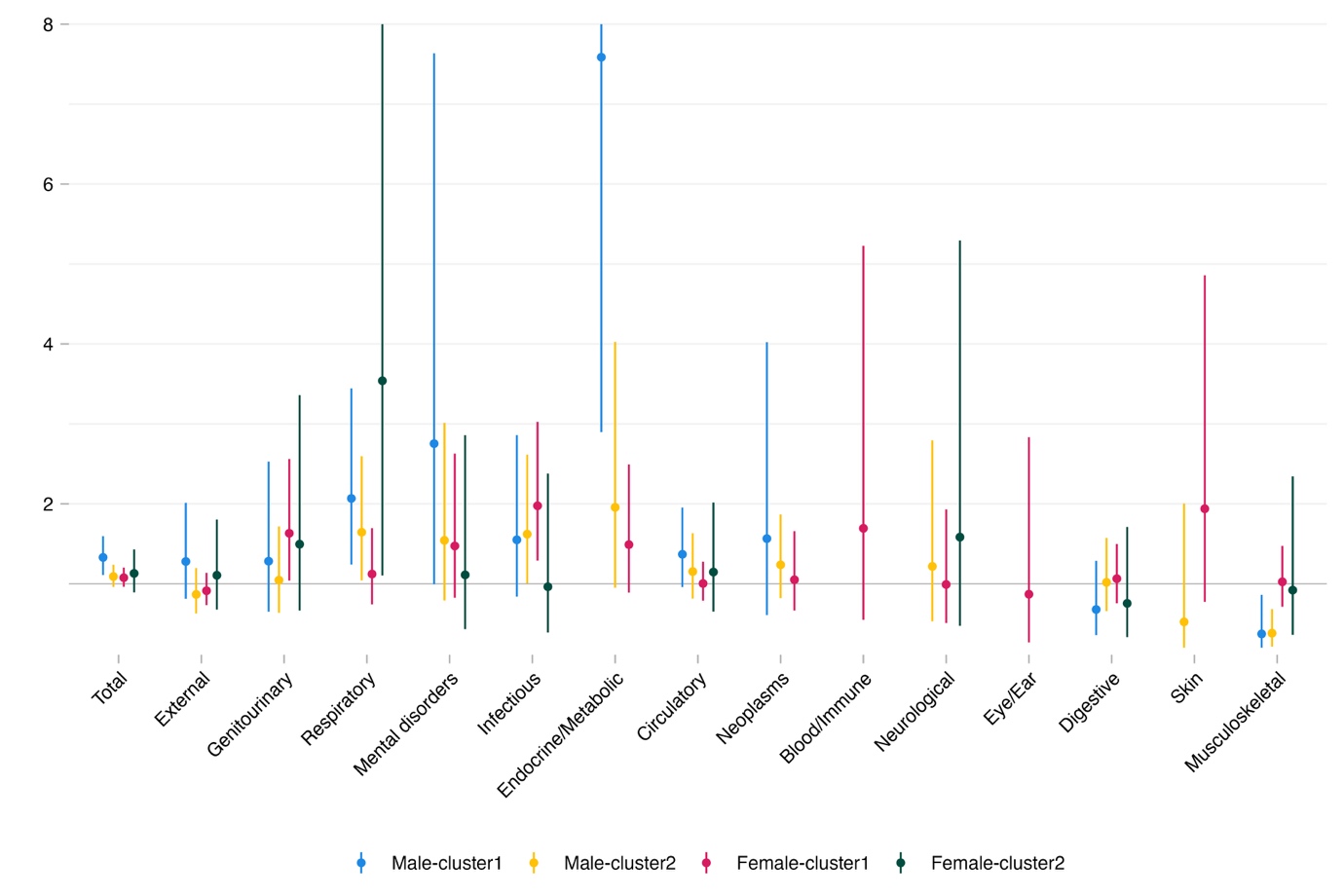


The characteristics of each cluster are:

- Male-Cluster 1 (n=2,665): Coronary heart disease and heart failure are the most prominent conditions in this group (around 60-80%), accompanied by moderate levels of cognitive impairment and diabetes. This pattern suggests a cardiovascular-dominant cluster with secondary metabolic and cognitive components.
- Male-Cluster 2 (n=5,762): This larger cluster presents a more even distribution across health conditions, with a moderate peak in cognitive impairment (~40%). Overall, it reflects a mixed-risk group with a generally lower overall disease burden.
- Female-Cluster 1 (n=8,732): This group shows generally low prevalence across most conditions, with only modest elevations in cognitive impairment (approximately 40%). The pattern suggests a broadly distributed health profile without a dominant condition, indicating a relatively lower-risk group.
- Female-Cluster 2 (n=1,851): There is a pronounced spike in cognitive impairment, dementia and Alzheimer’s (approaching 80-100%), indicating a subgroup with substantial neurological and cognitive burden. Other conditions occur at lower rates.

Supplementary Figure S4. Sensitivity analysis with different knot positions for sociodemographic factors and primary diagnosis at discharge**.** Cumulative relative risks (RR) and 95% confidence intervals (CI) were calculated for the 99th temperature percentile relative to the minimum hospitalisation risk percentile (MHP). The MHP was determined specifically for each subgroup. Results from the main model are shown in red.


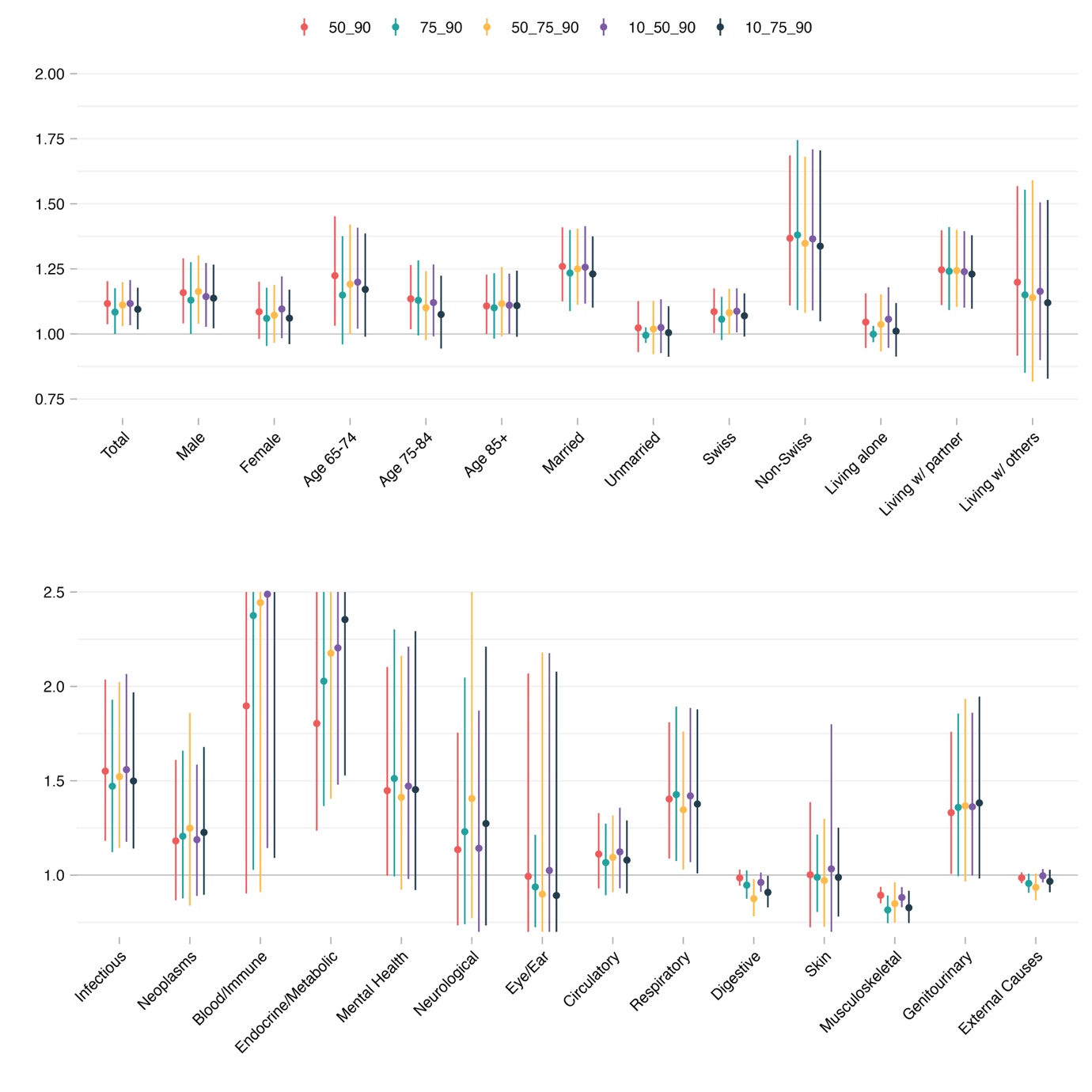

Supplement: dyag044_Supplementary_Data [file dyag044_supplementary_data.zip › 26-Mar-2026_072404_ije-2025-05-0985-File007.docx]
